# Priming the pump? Evaluating the effect of multiple intermittent theta burst sessions on cortical excitability in a nonhuman primate model

**DOI:** 10.1016/j.brs.2022.04.004

**Published:** 2022-04-07

**Authors:** Colleen A. Hanlon, Hilary R. Smith, Phillip M. Epperly, Miracle Collier, Lindsey K. Galbo, Paul W. Czoty

**Affiliations:** Department of Cancer Biology, Wake Forest School of Medicine, Winston-Salem, NC, USA; Department of Physiology & Pharmacology, Wake Forest School of Medicine, Winston-Salem, NC, USA; Department of Physiology & Pharmacology, Wake Forest School of Medicine, Winston-Salem, NC, USA; Department of Physiology & Pharmacology, Wake Forest School of Medicine, Winston-Salem, NC, USA; Department of Physiology & Pharmacology, Wake Forest School of Medicine, Winston-Salem, NC, USA; Department of Physiology & Pharmacology, Wake Forest School of Medicine, Winston-Salem, NC, USA; Department of Physiology & Pharmacology, Wake Forest School of Medicine, Winston-Salem, NC, USA

Leveraging preclinical knowledge regarding the role of hippocampal theta burst stimulation in learning and memory, in 2005 Huang and colleagues were the first to apply an intermittent bursting form of transcranial magnetic stimulation (theta burst stimulation, iTBS) to the human cortex [1]. Intermittent TBS increased cortical excitability in a manner that was more temporally efficient than non-bursting stimulation protocols. This manuscript has now been cited more than 3100 times and TBS may now be the dominant stimulation protocol of interest in the TMS field. It is currently used as a research tool and as a clinical treatment protocol for depression.

While the translational relevance and temporal efficiency of TBS are powerful, there are many unanswered questions regarding the inter-individual variability in response to TBS. There have been numerous studies demonstrating a high degree of within- and between-subject variance in changes in cortical excitability following a single session of iTBS [2–4]. While the brain stimulation field has begun to look deeper into biological factors that may explain response variability in humans, there is growing concern that, on a fundamental level, iTBS may not be a reliable tool to increase cortical excitability.

This study was designed to address this gap in our knowledge through a nonhuman primate model. Nonhuman primates provide a complementary experimental system with neuroanatomical similarity to humans, yet amenable to more controlled experimental designs. Using a cohort of 6 animals, we recently demonstrated that it is possible to collect motor evoked potentials and recruitment curves with high test-retest reliability using standard figure-of-8 coils in sedated NHPs [5].

The primary hypothesis of this study was that a single session of iTBS (600 pulses), does in fact, increase motor cortical excitability. A secondary hypothesis was that multiple TBS sessions induce a metaplasticity state wherein one iTBS session influences the next iTBS session. To test this hypothesis we evaluate the effect of 3 sessions of iTBS (10–15-min between-session interval) on cortical excitability in a cohort of 12 sedated NHPs.

## Methods:

The Wake Forest University Animal Care and Use Committee approved Procedures. Data were acquired from 12 male cynomolgous macaques (14.0 ± 1.2yrs; December 2020–January 2021). Coregistration and neuronavigation: A T1-weighted MRI scan from each animal was imported into the neuronavigation system (Brainsight 2.4, Rogue Research Inc., Canada), Scalp-to-cortex distance was measured (25.95 ± 11.35 mm; 9.2–43.4). EMG: Monkeys were sedated (5–10 mg/kg ketamine) followed by inhaled isoflurane (1.0–1.5% to effect). Once the animal was in place (supine, head and chest elevated approximately 45°), the forearm was shaved. Disposable surface electrodes (24mm Covidien) were placed over the right flexor digitorum. The EMG signal was recorded at 10240 Hz with a 5–3000Hz bandpass filter (EMG Pod, Rogue Research Inc., Canada). TMS setup: The scalp position associated with the largest MEP was marked as a target (D70 Air Film Coil, Magstim Super Rapid^2^, Welcony, UK). TMS experiment: Three sessions of iTBS (iTBS 1, 2 & 3) were delivered (600 pulses/session, 3 pulses/burst, 10.6-s inter train interval, 64% machine output (MO) (see supplement for parameter rationale). A 2 minute sampling period took place before and after each iTBS session (5 pulses @ 80, 90, 75, 85, 70, 95% MO delivered in that order). The peak-to-peak amplitude of the unrectified EMG signal was computed in the 50-ms response window beginning 10 ms after the TMS pulse. There was a 10 min break between each sampling interval (2 min) such that the average time between iTBS1–2 and iTBS 2–3 was 15.2 ± 0.3min. Analysis: A linear mixed model was constructed (repeated factors: time, trial, intensity; covariate: scalp-cortex distance; dependent measure: MEP; SPSS 26.0, IBM Statistics). Estimated marginal means were calculated for all fixed effects and subsequent pairwise comparisons were Bonferroni corrected.

## Results:

**Fig. 1.**
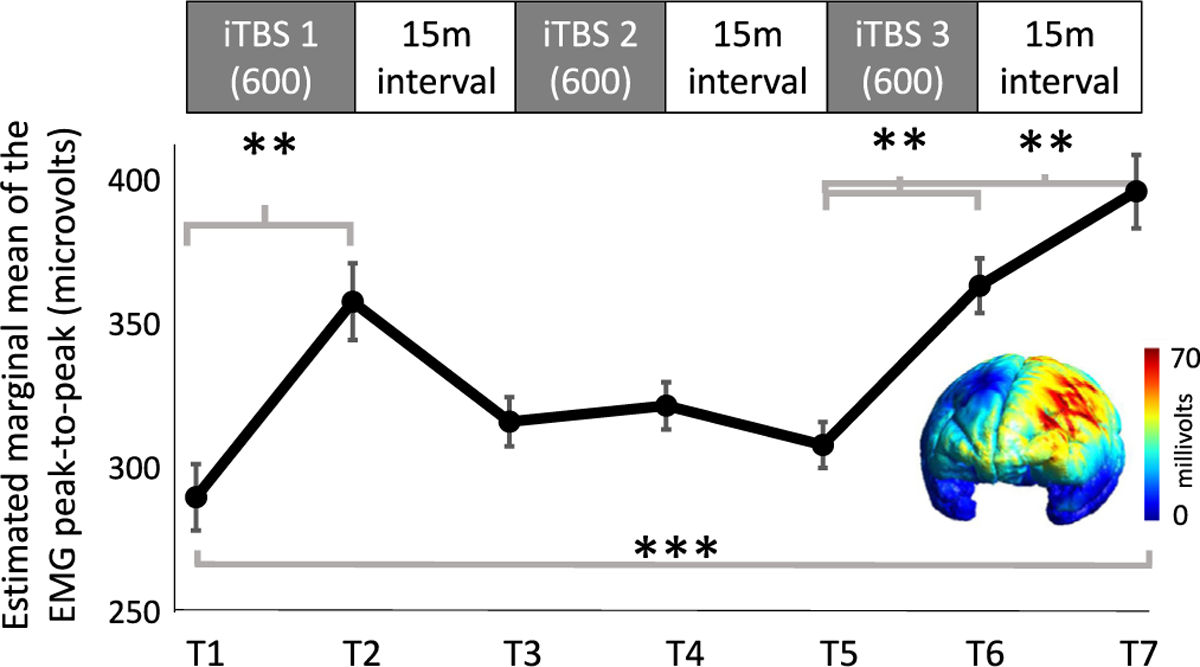
There was a significant effect of time (shown here; F(6,298) = 12.25, p < 0.001) and intensity (F(5,390) = 240.65, p < 0.001). Bonferroni corrected pairwise comparisons of time demonstrated that there was a significant increase in MEP immediately after the first session of iTBS (T1-T2; mean difference (MD)± standard error (SEM) = 62.9 ± 15.75μV p = 0.002) but this did not remain after the interval. There was also a significant increase in MEP immediately after the third session of iTBS (T5-T6; 51.3 ± 10.6μV p < 0.001) which remained elevated after the interval (T5-T7; 81.7 ± 13.3μV p < 0.001). Overall there was a significant increase in MEP after this 3 session protocol that lasted after the third iTBS session (T1-T7; 98.5 ± 15.5μV p < 0.001). Standard error shown here. See supplemental data for 95% confidence intervals and full output of the linear mixed model. An inset of the estimated electric field model for a rhesus macaque using the iTBS parameters is shown wherein the colormap corresponds to 0–70 mV (SIMNIBS).

There was an effect of time (Fig. 1; F(6,298) = 12.25, p < 0.001) and intensity (Supplemental data, F(5,390) = 240.65, p < 0.001) on MEP. MEP amplitude increased immediately after iTBS 1 (T1-T2; mean difference (MD) ± standard error (SEM) = 62.9 ± 15.75 μV, p = 0.002) and decayed during the 15-min interval. There was a significant increase in MEP immediately after iTBS 3 (T5-T6; 51.3 ± 10.6 μV, p < 0.001) which remained elevated (T5-T7; 81.7 ± 13.3 μV, p < 0.001). Overall there was a significant increase in MEP after this 3-session protocol that remained elevated after iTBS 3 (T1-T7; 98.5 ± 15.5 μV p < 0.001). Full output of the model and relevant comparisons are present in the supplementary data.

## Summary:

This study demonstrates that, in a cohort of 12 NHPs, 600 pulses of intermittent TBS reliably increases motor evoked potential - providing an important translational bridge between electrophysiological studies in rodents and applied studies in humans. The NHPs in this study were all sedated (which limits the direct translational impact to humans). This is also an important strength, however, as it reveals that in a controlled brain state 600 pulses of iTBS reliably increases cortical excitability. This is consistent with an observation by Papazachariadis and colleagues (2014) that 600 pulses of iTBS can increase somatosensory evoked potentials as well as the power of theta and gamma oscillations in sedated NHPs [10]. Additionally, our data suggest that iTBS may display metaplasticity properties, wherein the threshold, direction, and agnitude of change is influenced/primed by previous sessions [6]. This adds to a growing body of research demonstrating the impact of priming on TBS-induced cortical excitability change [7–9]. The details of the timecourse, however, will need to be clarified in future experiments. We acknowledge barriers that limit generalizability of this NHP model to human literature. The most potent is likely the use of sedation in these animals. The controlled state of the animal, however, led to low variability in MEPs - supporting the suggestion that variability in “brain state” may be one of the most influential factors contributing to TBS variability in human subjects. As with all science, the results of this study are limited to the specific protocol tested in this study. We believe that data from these 12 NHPs adds a valuable and complementary contribution to our knowledge regarding the ability of iTBS to increase cortical excitability when animals are in a controlled brain state.

## Supplementary Material

S1

## Appendix A. Supplementary data

Supplementary data to this article can be found online at https://doi.org/10.1016/j.brs.2022.04.004.
