# Supplementary material for: Priming the pump? Evaluating the effect of multiple intermittent theta burst sessions on cortical excitability in a nonhuman primate model
Source: Brain Stimul. Author manuscript; Available in PMC 2026 Jul 1. (PMC13321387; doi:10.1016/j.brs.2022.04.004)
Supplement: S1 [file NIHMS2041041-supplement-S1.docx]

**Intermittent TBS increases motor cortical excitability: insight from a nonhuman primate model**

**Supplementary Data:**

**Subjects:** Twelve socially housed male Cynomolgus monkeys (Macaca fascicularis) between 12-14 years of age, and between 5.84-8.49kg served as subjects. Monkeys had a 3-month history of drinking ethanol in a controlled setting.

**Anesthesia Procedure:** The animals were sedated with ketamine (10mg/kg, i.m.) and moved to the procedure room. Once in the procedure room animals were either intubated and put on a ventilator or had a face mask placed. They were then maintained at ~1.5% isoflurane for the remainder of the experiment.

**Study design:** . EMG data was collected at 7 time points from 12 non-human primates before and after 3 sessions of intermittent theta burst stimulation (iTBS, 600 pulses, 64% machine output (MO), 2 sec on, 8 sec off; Magstim Rapid D70) delivered to the left primary motor cortex (MRI-assisted neuronavigation; Rogue Research). In a previous publication (Hanlon et al 2020) we demonstrated that the average motor threshold of macaques with a similar anesthesia profile and similar scalp to cortex distance was 74% machine output. iTBS typically delivered at 80% of an active motor threshold, but is occasionally given at higher doses, up to 110% resting motor threshold. 80% of the original average RMT in this nonhuman primate model would be 60% machine output. We chose to use 64% machine output as it would likely meet the minimum of 80% motor threshold for each animal and it was the maximum that the coil could deliver. Scalp-to-cortex distance was measured dynamically viea the neuronavigationsystem and was used as a regressor in the data analysis. Approximately fifteen minutes elapsed between each iTBS session. This included a ten minute pause (wherein the research team stayed in the room with the animal while MEP ws recorded continuously, as well as a sampling perior before and after each iTBS period (2 minutes each). At each sampling period 5 pulses of TMS were delivered in a random block order at 6 intensities (70, 75, 80, 85, 90, 95% MO; 30 pulses/timepoint/animal).

**Analysis.** A linear mixed model was used to evaluate the repeated effect of iTBS session on MEP (time, intensity, trial as repeated measures; scalp-cortex distance as covariate; MEP dependent measure; SPSS).: Scalp to cortex distance was used as a covariate in the mixed model. The results of the Fixed Effects analysis, Estimated Marginal Means, and Pairwise comparisons for Time and Intensity are shown below.

**Linear Mixed Model:**

| **Type III Tests of Fixed Effects^a^** | | | | |
| --- | --- | --- | --- | --- |
| Source | Numerator df | Denominator df | F | Sig. |
| Intercept | 1 | 1167.948 | 5799.433 | 0.000 |
| time | 6 | 298.786 | 12.250 | 0.000 |
| intensity | 5 | 390.085 | 240.659 | 0.000 |

Estimated Marginal Means: Time

| **Estimates^a^** | | | | | |
| --- | --- | --- | --- | --- | --- |
| time | Mean | Std. Error | df | 95% Confidence Interval |  |
|  |  |  |  | Lower Bound | Upper Bound |
| 1.00 | 300.058 | 10.702 | 255.974 | 278.982 | 321.134 |
| 2.00 | 363.004 | 12.317 | 177.798 | 338.697 | 387.311 |
| 3.00 | 324.378 | 7.893 | 260.665 | 308.836 | 339.920 |
| 4.00 | 329.511 | 7.595 | 300.494 | 314.565 | 344.457 |
| 5.00 | 316.835 | 7.410 | 285.319 | 302.249 | 331.420 |
| 6.00 | 368.149 | 8.796 | 238.559 | 350.822 | 385.477 |
| 7.00 | 398.512 | 11.804 | 245.242 | 375.261 | 421.762 |
| a. Dependent Variable: MEP. |  |  |  |  |  |

Pairwise Comparisons: Time

| **Pairwise Comparisons^a^** | | | | | | | |
| --- | --- | --- | --- | --- | --- | --- | --- |
| (I) time |  | Mean Difference (I-J) | Std. Error | df | Sig.^c^ | 95% Confidence Interval for Difference^c^ |  |
|  |  |  |  |  |  | Lower Bound | Upper Bound |
| 1.00 | 2.00 | -62.946^*^ | 15.751 | 283.052 | 0.002 | -111.235 | -14.658 |
|  | 3.00 | -24.320 | 12.806 | 364.232 | 1.000 | -63.499 | 14.859 |
|  | 4.00 | -29.453 | 12.641 | 357.718 | 0.428 | -68.135 | 9.228 |
|  | 5.00 | -16.777 | 12.488 | 390.973 | 1.000 | -54.966 | 21.413 |
|  | 6.00 | -68.091^*^ | 13.449 | 427.649 | 0.000 | -109.197 | -26.986 |
|  | 7.00 | -98.454^*^ | 15.532 | 438.929 | 0.000 | -145.918 | -50.989 |
| 2.00 | 1.00 | 62.946^*^ | 15.751 | 283.052 | 0.002 | 14.658 | 111.235 |
|  | 3.00 | 38.626 | 14.078 | 232.227 | 0.138 | -4.621 | 81.873 |
|  | 4.00 | 33.493 | 13.965 | 245.553 | 0.362 | -9.379 | 76.365 |
|  | 5.00 | 46.170^*^ | 13.816 | 258.412 | 0.020 | 3.776 | 88.564 |
|  | 6.00 | -5.145 | 14.680 | 323.235 | 1.000 | -50.100 | 39.810 |
|  | 7.00 | -35.507 | 16.590 | 368.237 | 0.693 | -86.263 | 15.248 |
| 3.00 | 1.00 | 24.320 | 12.806 | 364.232 | 1.000 | -14.859 | 63.499 |
|  | 2.00 | -38.626 | 14.078 | 232.227 | 0.138 | -81.873 | 4.621 |
|  | 4.00 | -5.133 | 10.204 | 348.484 | 1.000 | -36.363 | 26.097 |
|  | 5.00 | 7.543 | 10.023 | 351.870 | 1.000 | -23.129 | 38.216 |
|  | 6.00 | -43.771^*^ | 11.094 | 403.958 | 0.002 | -77.689 | -9.853 |
|  | 7.00 | -74.134^*^ | 13.634 | 356.051 | 0.000 | -115.855 | -32.412 |
| 4.00 | 1.00 | 29.453 | 12.641 | 357.718 | 0.428 | -9.228 | 68.135 |
|  | 2.00 | -33.493 | 13.965 | 245.553 | 0.362 | -76.365 | 9.379 |
|  | 3.00 | 5.133 | 10.204 | 348.484 | 1.000 | -26.097 | 36.363 |
|  | 5.00 | 12.677 | 9.505 | 339.387 | 1.000 | -16.418 | 41.771 |
|  | 6.00 | -38.638^*^ | 10.802 | 370.260 | 0.008 | -71.684 | -5.592 |
|  | 7.00 | -69.000^*^ | 13.377 | 349.952 | 0.000 | -109.939 | -28.062 |
| 5.00 | 1.00 | 16.777 | 12.488 | 390.973 | 1.000 | -21.413 | 54.966 |
|  | 2.00 | -46.170^*^ | 13.816 | 258.412 | 0.020 | -88.564 | -3.776 |
|  | 3.00 | -7.543 | 10.023 | 351.870 | 1.000 | -38.216 | 23.129 |
|  | 4.00 | -12.677 | 9.505 | 339.387 | 1.000 | -41.771 | 16.418 |
|  | 6.00 | -51.315^*^ | 10.599 | 251.473 | 0.000 | -83.846 | -18.783 |
|  | 7.00 | -81.677^*^ | 13.262 | 300.970 | 0.000 | -122.312 | -41.042 |
| 6.00 | 1.00 | 68.091^*^ | 13.449 | 427.649 | 0.000 | 26.986 | 109.197 |
|  | 2.00 | 5.145 | 14.680 | 323.235 | 1.000 | -39.810 | 50.100 |
|  | 3.00 | 43.771^*^ | 11.094 | 403.958 | 0.002 | 9.853 | 77.689 |
|  | 4.00 | 38.638^*^ | 10.802 | 370.260 | 0.008 | 5.592 | 71.684 |
|  | 5.00 | 51.315^*^ | 10.599 | 251.473 | 0.000 | 18.783 | 83.846 |
|  | 7.00 | -30.362 | 14.103 | 311.622 | 0.674 | -73.563 | 12.838 |
| 7.00 | 1.00 | 98.454^*^ | 15.532 | 438.929 | 0.000 | 50.989 | 145.918 |
|  | 2.00 | 35.507 | 16.590 | 368.237 | 0.693 | -15.248 | 86.263 |
|  | 3.00 | 74.134^*^ | 13.634 | 356.051 | 0.000 | 32.412 | 115.855 |
|  | 4.00 | 69.000^*^ | 13.377 | 349.952 | 0.000 | 28.062 | 109.939 |
|  | 5.00 | 81.677^*^ | 13.262 | 300.970 | 0.000 | 41.042 | 122.312 |
|  | 6.00 | 30.362 | 14.103 | 311.622 | 0.674 | -12.838 | 73.563 |
| Based on estimated marginal means | | | | | | | |
| *. The mean difference is significant at the .05 level. | | | | | | | |
| a. Dependent Variable: MEP.  c. Adjustment for multiple comparisons: Bonferroni. | | | | | | | |

Estimated Marginal Means: Intensity:

| **Estimates^a^** | | | | | |
| --- | --- | --- | --- | --- | --- |
| intensity | Mean | Std. Error | df | 95% Confidence Interval |  |
|  |  |  |  | Lower Bound | Upper Bound |
| 70.00 | 144.627 | 6.425 | 278.314 | 131.980 | 157.274 |
| 75.00 | 213.083 | 6.917 | 326.490 | 199.475 | 226.691 |
| 80.00 | 278.431 | 6.563 | 372.460 | 265.525 | 291.336 |
| 85.00 | 357.091 | 7.915 | 315.099 | 341.518 | 372.664 |
| 90.00 | 476.381 | 13.125 | 297.191 | 450.552 | 502.210 |
| 95.00 | 587.914 | 16.709 | 306.810 | 555.036 | 620.793 |
| a. Dependent Variable: MEP. |  |  |  |  |  |

Pairwise comparisons: Intensity:

| **Pairwise Comparisons^a^** | | | | | | | |
| --- | --- | --- | --- | --- | --- | --- | --- |
| (I) intensity |  | Mean Difference (I-J) | Std. Error | df | Sig.^c^ | 95% Confidence Interval for Difference^c^ |  |
|  |  |  |  |  |  | Lower Bound | Upper Bound |
| 70.00 | 75.00 | -68.456^*^ | 9.263 | 410.678 | 0.000 | -95.806 | -41.107 |
|  | 80.00 | -133.804^*^ | 8.999 | 502.923 | 0.000 | -160.345 | -107.263 |
|  | 85.00 | -212.464^*^ | 10.042 | 596.610 | 0.000 | -242.058 | -182.870 |
|  | 90.00 | -331.754^*^ | 14.530 | 453.921 | 0.000 | -374.631 | -288.877 |
|  | 95.00 | -443.287^*^ | 17.834 | 407.414 | 0.000 | -495.943 | -390.632 |
| 75.00 | 70.00 | 68.456^*^ | 9.263 | 410.678 | 0.000 | 41.107 | 95.806 |
|  | 80.00 | -65.348^*^ | 9.099 | 435.445 | 0.000 | -92.205 | -38.491 |
|  | 85.00 | -144.008^*^ | 10.061 | 524.883 | 0.000 | -173.674 | -114.341 |
|  | 90.00 | -263.298^*^ | 14.596 | 430.909 | 0.000 | -306.380 | -220.216 |
|  | 95.00 | -374.831^*^ | 17.909 | 401.400 | 0.000 | -427.714 | -321.949 |
| 80.00 | 70.00 | 133.804^*^ | 8.999 | 502.923 | 0.000 | 107.263 | 160.345 |
|  | 75.00 | 65.348^*^ | 9.099 | 435.445 | 0.000 | 38.491 | 92.205 |
|  | 85.00 | -78.660^*^ | 9.831 | 462.450 | 0.000 | -107.666 | -49.653 |
|  | 90.00 | -197.950^*^ | 14.438 | 402.874 | 0.000 | -240.581 | -155.318 |
|  | 95.00 | -309.483^*^ | 17.797 | 383.059 | 0.000 | -362.050 | -256.917 |
| 85.00 | 70.00 | 212.464^*^ | 10.042 | 596.610 | 0.000 | 182.870 | 242.058 |
|  | 75.00 | 144.008^*^ | 10.061 | 524.883 | 0.000 | 114.341 | 173.674 |
|  | 80.00 | 78.660^*^ | 9.831 | 462.450 | 0.000 | 49.653 | 107.666 |
|  | 90.00 | -119.290^*^ | 15.126 | 423.741 | 0.000 | -163.942 | -74.638 |
|  | 95.00 | -230.824^*^ | 18.320 | 396.865 | 0.000 | -284.923 | -176.724 |
| 90.00 | 70.00 | 331.754^*^ | 14.530 | 453.921 | 0.000 | 288.877 | 374.631 |
|  | 75.00 | 263.298^*^ | 14.596 | 430.909 | 0.000 | 220.216 | 306.380 |
|  | 80.00 | 197.950^*^ | 14.438 | 402.874 | 0.000 | 155.318 | 240.581 |
|  | 85.00 | 119.290^*^ | 15.126 | 423.741 | 0.000 | 74.638 | 163.942 |
|  | 95.00 | -111.534^*^ | 21.140 | 514.694 | 0.000 | -173.876 | -49.191 |
| 95.00 | 70.00 | 443.287^*^ | 17.834 | 407.414 | 0.000 | 390.632 | 495.943 |
|  | 75.00 | 374.831^*^ | 17.909 | 401.400 | 0.000 | 321.949 | 427.714 |
|  | 80.00 | 309.483^*^ | 17.797 | 383.059 | 0.000 | 256.917 | 362.050 |
|  | 85.00 | 230.824^*^ | 18.320 | 396.865 | 0.000 | 176.724 | 284.923 |
|  | 90.00 | 111.534^*^ | 21.140 | 514.694 | 0.000 | 49.191 | 173.876 |
| Based on estimated marginal means | | | | | | | |
| *. The mean difference is significant at the .05 level. | | | | | | | |
| a. Dependent Variable: MEP. | | | | | | | |
| c. Adjustment for multiple comparisons: Bonferroni. 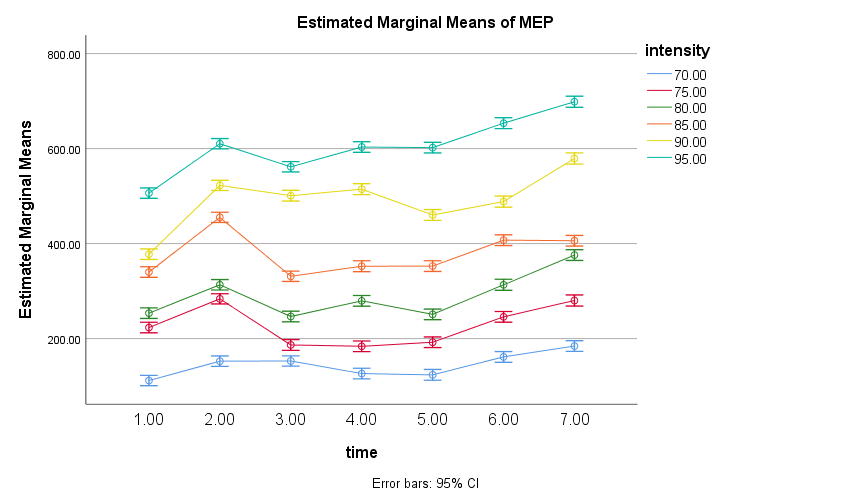 | | | | | | | |
